# Anti-Vaccinationists and Modified Smallpox

**Published:** 1909-08-21

**Authors:** Joseph P. Swan

**Affiliations:** 11 Scarisbrick Street, Southport


### Editor's Letter-Box.

### ANTI-VACCINATIONISTS AND MODIFIED SMALLPOX.

*To the Editor of* The Hospital.

Sir,—Adverting to the article in your issue of July 31, headed "Anti-vaccinationists and Modified Smallpox," I should be glad if you would state in your columns :

1. Where in the *Vaccination Inquirer* has serum or vaccine therapy been described as "filthy iniquity," "loathsome art," or "unclean fraud."

2. Also where is it asserted that vaccination is upheld by the medical profession solely because it affords fees.

3. When you say that "bacteria are no more and no less unclean than the yeast with which bread is made," do you mean this remark to apply to the living bacteria of specific diseases?

4. If vaccination is the "intentional transmission of disease," how can it be honestly compared with soup, bread, and beef-steak?

5. How can the adult population of Leicester be "the least efficiently vaccinated part of the population" in view of the fact that practically the whole of the child population is unvaccinated?

6. If vaccination has hitherto been practised only as a "system of empiricism," don't you think that even language of the anti-vaccinationist is inadequate to denounce its legal enforcement?

Yours faithfully,

Joseph P. Swan.

11 Scarisbrick Street, Southport, August 5, 1909.

[Mr. Swan's questions can be very briefly answered. (1) The quotations are from the copy of the *Vaccination Inquirer* forwarded by Mr. Swan to the offices of The Hospital. (2) The same applies to this question. (3) The bacterium of a specific disease, whether alive or dead, is strictly comparable with any other unicellular organism, such as yeast. Neither is in the least "unclean" in any sense of that term which we can admit; the lesions produced in a susceptible animal by the invasion of micro-organisms are, of course, to be distinguished from the organisms themselves. (4) Vaccination was not compared with soup, bread, or beef-steak; there is no comparison possible between concrete substances like these and the act of vaccination. The comparison made was between culture media and soup, and between serum and beef-steak; and these comparisons are strictly accurate. (5) The answer to this question is contained in the paragraphs which Mr. Swan marked for us in his paper. Since ten or twelve years is, on the average, the period during which vaccination protects against smallpox, then an adult population, many of whom have never been vaccinated, and very few of whom have been re-vaccinated, may reasonably be described as less efficiently vaccinated than a child population a certain proportion of whom are adequately protected by vaccination. (6) Does Mr. Swan fully realise the meaning of the word empiricism? Until the last generation practically all medicine and surgery has been empirical, and much of it is so still. A remedy or a system which has been empirically tested and found useful is not a thing to abandon until something better is procured. No one proposes to abandon the quinine treatment of malaria, which is empiricism pure and simple if anything ever has been, until some better therapeutic measure is brought forward; some day, possibly, a cure for this disease based on the biology of the specific plasmodium may be introduced, but even then it will have to be empirically tested before it can be adopted. We cannot understand, much less sympathise with, the attitude of mind which denounces the enforcement of a measure found to be empirically useful, and we do not admit for a moment that there is anything in the least ignoble about empiricism. —Editor, The Hospital.]

